# Association between Asthma and Periodontitis

**DOI:** 10.3390/diagnostics13243637

**Published:** 2023-12-10

**Authors:** Sung Joon Park, Hahn Jin Jung, Min Woo Park, Hyo Geun Choi, Heejin Kim, Jee Hye Wee

**Affiliations:** 1 Department of Otorhinolaryngology-Head and Neck Surgery, Chung-Ang University Gwangmyeong Hospital, Chung-Ang University College of Medicine, Seoul 06974, Republic of Korea; hypocratis@gmail.com; 2 Department of Otorhinolaryngology-Head and Neck Surgery, Chungbuk National University College of Medicine, Chungbuk National University Hospital, Cheongju 28644, Republic of Korea; hahnjin2@naver.com; 3 Department of Otorhinolaryngology-Head and Neck Surgery, Kangdong Sacred Heart Hospital, Seoul 05355, Republic of Korea; subintern@naver.com; 4 Department of Otorhinolaryngology-Head and Neck Surgery, Mdanalytics, Suseoseoulent Clinic, Seoul 06349, Republic of Korea; pupen@naver.com; 5 Department of Otorhinolaryngology-Head and Neck Surgery, Hallym University Sacred Heart Hospital, Hallym University College of Medicine, Anyang 14068, Republic of Korea; heejin5020@daum.net

**Keywords:** asthma, periodontitis, chronic disease

## Abstract

The current study aimed to investigate the association between asthma and periodontitis in the Korean adult population. Data from the Korean Genome and Epidemiology Study Health Examinees between 2004 and 2016 were considered. Of the 173,209 participants, 2521 asthmatic and 132,806 control participants were selected. The participants were categorized according to their current status of asthma, as ‘well-controlled’, ‘being treated’, and ‘not being treated’. The prevalence of periodontitis was found to be significantly higher in the participants with asthma (13.1%) than in the controls (7.3%). In the fully adjusted model, the patients with asthma had a higher odds ratio (OR = 1.79, 95% confidence interval [CI] = 1.59–2.02, *p* < 0.001) for periodontitis than those without asthma. The results were consistent across all the age and sex subgroups. The adjusted ORs for periodontitis were 2.15 (95% CI = 1.68–2.76, *p* < 0.001) in the ‘well-controlled’ asthma group, 1.44 (95% CI = 1.16–1.78, *p* < 0.001) in the ‘being treated’ asthma group, and 1.86 (95% CI = 1.55–2.22, *p* < 0.001) in the ’not being treated’ asthma group compared to the control group. Overall, we found asthma to be associated with periodontitis in Korean adults, and the participants with well-controlled asthma had the highest ORs for periodontitis.

## 1. Introduction

Asthma is a chronic respiratory disease characterized by reversible airway obstruction that usually results from allergic reactions or hypersensitivity [1]. Asthma was responsible for 21.6 million disability-adjusted life years in 2019, which was 20.8% of the total disability-adjusted life years due to chronic respiratory diseases. The incidence of asthma in 2019 was 37 million, which represented a global increase of 13.0% since 2010 [2]. The direct and indirect costs of asthma in Korea are approximately USD 553.9 million and USD 92.0 million, respectively [3].

Patients with asthma have been reported to have more comorbidities than those without asthma. In a meta-analysis, the prevalence of cardiovascular diseases (odds ratio [OR] = 1.90, 95% confidence interval [CI] = 1.70–2.14), cerebrovascular diseases (OR = 1.44, 95% CI = 1.29–1.60), obesity (OR = 1.51, 95% CI = 1.14–2.01), hypertension (OR = 1.66, 95% CI = 1.47–1.88), diabetes (OR = 1.25, 95% CI = 1.08–1.44), other metabolic and endocrine diseases (OR = 1.60, 95% CI = 1.40–1.83), psychiatric and neurological diseases (OR = 1.62, 95% CI = 1.44–1.82), gut and urinary diseases (OR = 1.91, 95% CI = 1.47–2.49), cancer (OR = 1.17, 95% CI = 1.10–1.25), and respiratory diseases (OR = 5.60, 95% CI = 4.22–7.44) are significantly higher in patients with asthma in comparison with controls [4]. Comorbidities can complicate the clinical care of asthma patients in various ways. Therefore, assessment of comorbidities is mandatory in asthma management.

Periodontitis is a chronic oral inflammation and infection characterized by the loss of periodontal tissue support [5]. Its pathophysiology has been characterized at the molecular level, leading to the activation of host-derived proteinases that facilitate the loss of marginal periodontal ligament fibers, apical migration of the junctional epithelium, and the spread of the bacterial biofilm along the root surface [6]. As successful management of periodontitis can be challenging to control due to its chronic nature and complex contributing factors [7], several studies emphasize the significance of preventing periodontal disease [8,9]. Moreover, its effects are not limited to the periodontal tissue but spread throughout the vascular system, increasing the level of systemic inflammation, and may be related to other systemic diseases [10]. A systematic review and meta-analysis have shown that the pooled prevalence of apical periodontitis is 52% at the individual level (95% CI = 42–56%, I^2^ = 97.8%) and is higher in individuals with a systemic condition (63%, 95% CI = 56–69%, I^2^ = 89.7%) compared to healthy individuals (48%, 95% CI = 43–56%, I^2^ = 98.3%) [11]. Evidence is available on the relationship between periodontitis and systemic inflammatory diseases, such as diabetes mellitus, cardiovascular diseases, and respiratory diseases [10,12,13]. 

The results on the correlation between asthma and periodontitis have been conflicting. In a U.S. cross-sectional study, a significant inverse association was observed between self-reported asthma and periodontal disease (adjusted OR = 0.695, 95% CI = 0.564–0.857) [14]. The study explained that the protective effect against inflammation in asthma could be attributed to the regular use of anti-inflammatory medications by patients with asthma. Patients with periodontitis in Austria had a significantly lower prevalence of asthma than the general population (adjusted OR = 0.169, 95% CI = 0.106–0.270) [15]. Conversely, several studies have supported a link between asthma and periodontitis. A case–control study conducted in Brazil reported that periodontitis was associated with severe asthma (adjusted OR = 4.00, 95% CI = 2.26–7.10) [16]. A Taiwanese population study showed that patients with asthma had an increased risk of developing periodontal disease (adjusted hazard ratio = 1.18, 95% CI = 1.14–1.22) [17]. The authors emphasized that the heightened risk of periodontal disease in patients with asthma results from anti-asthmatic medications, especially inhaled corticosteroids. In a study using data from the Korea National Health and Nutrition Examination Survey VI-1, patients with current asthma conditions have been shown to be approximately five times more likely to be associated with periodontitis (adjusted OR = 5.36, 95% CI = 1.27–22.68) [18]. However, only 89 patients with asthma were included in that study.

Since both asthma and periodontitis are prevalent diseases, the relationship between the two could be informative for clinicians managing patients with asthma. In particular, periodontitis affects approximately 50% of the adult population worldwide, and its prevalence and severity increase with age, with the incidence rate reported steeply increasing in adults aged 30–40 years [19]. Therefore, we aimed to investigate the association between asthma and periodontitis in adults over 40 years of age. Additionally, we evaluated the association between asthma and periodontitis based on the status of asthma treatment. 

## 2. Materials and Methods

### 2.1. Study Population and Data Collection

The research was authorized by the ethics committee of Hallym University (2019-02-020); all the assessments adhered to the standards and rules set forth by the ethics committee. The requirement for written informed consent was exempted by the Institutional Review Board. This prospective cohort investigation utilized data from the Korean Genome and Epidemiology Study (KoGES) spanning from 2004 to 2016. A comprehensive depiction of the data was provided in a prior study [20]. The KoGES is a consortium project consisting of six prospective cohort studies (the population-based cohorts include the community-based KoGES Ansan and Ansung study, the urban community-based KoGES health examinee (HEXA) study, the rural community-based KoGES cardiovascular disease association study (CAVAS), and the KoGES gene–environment model studies including the KoGES twin and family study, the KoGES_immigrant study, and the KoGES_emigrant study). Within the Korean Genome and Epidemiology Study Consortium consisting of six cohort studies, we utilized data from the urban community-based KoGES HEXA study involving volunteers aged ≥ 40 years who visited the associated institutions, which are mainly general hospitals in the metropolitan areas and major Korean cities. The participants were recruited from the National Health Examinee Registry, which is part of the National Health Insurance Program. The dataset comprised baseline information collected in 39 cities from 2004 to 2013 and follow-up data from 2012 to 2016.

### 2.2. Participant Selection

**Figure 1:**
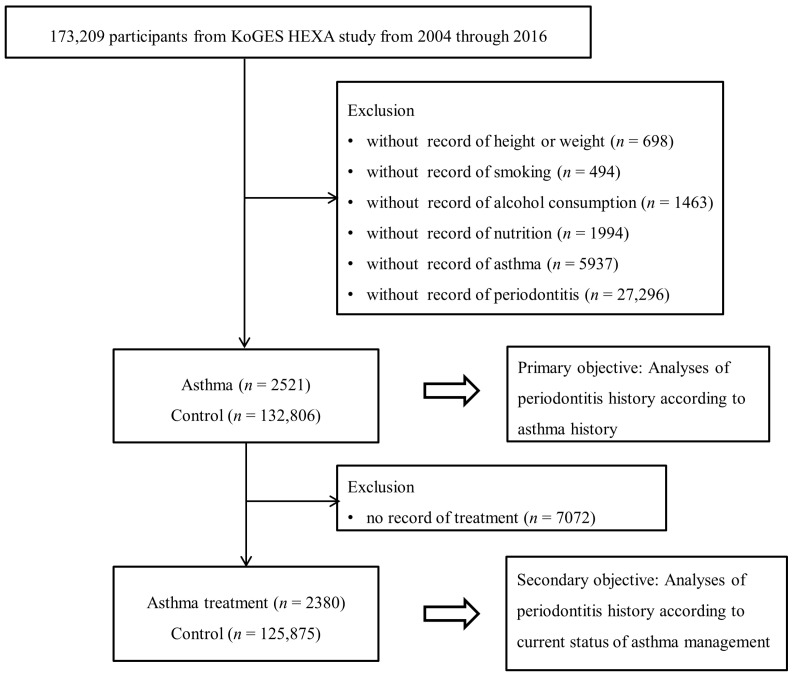
A schematic illustration of the participant selection process that was used in the present study.

Out of the 173,209 participants, those lacking records of height or weight (*n* = 698), smoking history (*n* = 494), alcohol consumption (*n* = 1463), nutrition (*n* = 1994), asthma (*n* = 5973), or periodontitis (*n* = 27,296) were excluded. Numerous participants were excluded due to the absence of a history of asthma and periodontitis; asthma was not surveyed in 2004, and periodontitis was not surveyed from 2004 to 2006. Eventually, 2521 participants with asthma and 132,806 controls (with non-asthma history) were chosen (Figure 1). Subsequently, we compared the history of periodontitis between the asthmatic and control participants (primary objective). Next, we examined the history of periodontitis according to the current status of asthma management (secondary objective). Of the participants, 7072 were excluded because of a lack of treatment records, since they had not been surveyed in 2008.

### 2.3. Survey

Trained interviewers inquired about the participants’ sociodemographic status, lifestyle, and past histories of diseases using a validated questionnaire, for example, “Have you ever been diagnosed with asthma?”. The participants were defined as asthma patients if they were previously diagnosed by a medical doctor. If the participants answered “no” to this question, they were defined as controls and included in the non-asthma group. Participants with asthma were further questioned about their present status of asthma management and were sorted into three categories; those informed by a medical doctor that medical treatment was unnecessary due to symptom resolution (well-controlled), those currently undergoing treatment (being treated), and those experiencing untreated symptoms (not being treated) [21]. The participants were also queried about their history of periodontitis, for example, “Have you ever been diagnosed with periodontitis?” [22].

According to their smoking history, the participants were classified as non-smokers (<100 cigarettes in their entire life), former smokers (quit more than one year ago), and current smokers. Alcohol consumption was divided into non-drinkers, former drinkers, and current drinkers. Nutritional intake (total calories [kcal/day], protein [g/day], fat [g/day], and carbohydrates [g/day]) was assessed using a food frequency questionnaire validated in a prior study [22]. Income group was stratified as non-respondent, low-income (<USD 2000 per month), middle-income (USD 2000–3999 per month), and high-income (≥USD 4000 per month), according to the household income. Body mass index (BMI) was computed in kg/m^2^ using health checkup data.

### 2.4. Statistical Analyses

Chi-square tests were employed to compare the rates of sex, income group, smoking status, and alcohol consumption. Independent t-tests were utilized to compare age, BMI, and nutritional intake.

As the primary goal of this study, a logistic regression model was employed to compute the OR of periodontitis associated with asthma. Both crude and adjusted models (considering age, sex, income group, BMI, smoking status, alcohol consumption, and nutritional intake, such as total calorie, protein, fat, and carbohydrate intake) were used. In the subgroup analyses according to age, the dividing point was set at the median age (<53 years old and ≥53 years old). For the secondary objective, the ORs of asthma treatment (‘well-controlled’, ‘being treated’, and ‘not being treated’, compared to control participants) for periodontitis were calculated using a logistic regression model.

Two-tailed analyses were carried out, and *p* values below 0.05 were deemed significant. The results were analyzed using SPSS (version 24.0; IBM, Armonk, NY, USA).

## 3. Results

The general characteristics of the participants varied between the asthmatic and control groups (Table 1). Compared with the control groups, the individuals with asthma were older, more prone to obesity, had lower incomes, were non-smokers, and abstained from alcohol. The prevalence of periodontitis was significantly higher in the patients with asthma (13.1%) than in the controls (7.3%) (*p* < 0.001).

The patients with asthma exhibited a higher adjusted OR for periodontitis (1.79, 95% CI = 1.59–2.02, *p* < 0.001) than those without asthma in the multiple logistic regression model (Table 2). In the subgroup analysis, the higher adjusted OR for periodontitis in the asthma group was consistent regardless of age and sex. The adjusted ORs were 2.17 (95% CI = 1.48–3.20) in men under 53 years old, 1.64 (95% CI = 1.27–2.12) in women under 53 years old, 1.87 (95% CI = 1.47–2.38) in men aged 53 and older, and 1.81 (95% CI = 1.51–2.16) in women aged 53 and older (all *p* < 0.05).

**Figure 2:**
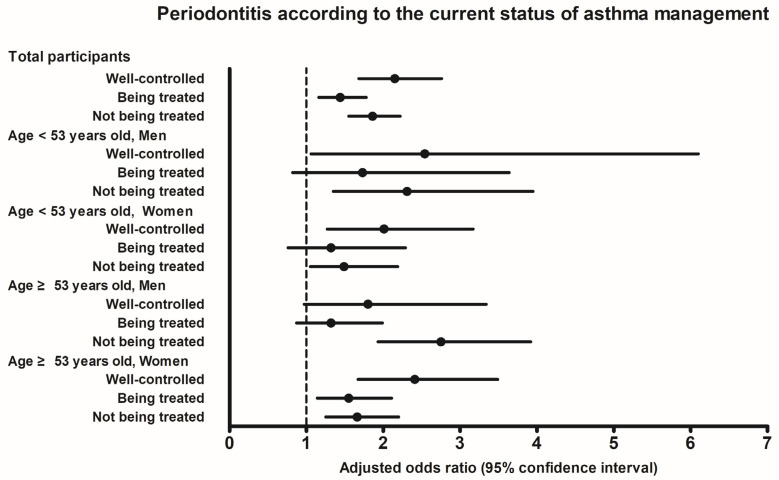
Adjusted odds ratios and 95% confidence interval for periodontitis according to the current status of asthma management.

Table 3 and Figure 2 show the ORs for periodontitis based on the current status of asthma management, categorized by age and sex. The adjusted OR for periodontitis was 2.15 (95% CI = 1.68–2.76) in the ‘well-controlled’ asthma group, 1.44 (95% CI = 1.16–1.78) in the ‘being treated’ asthma group, and 1.86 (95% CI = 1.55–2.22) in the ‘not being treated’ asthma group (all *p* < 0.001). Following stratification by age and sex, the ‘being treated’ asthma subgroup did not show statistical significance compared to the control in all the men and the <53-year-old women. On the other hand, the ‘well-controlled’ and ‘being treated’ asthma subgroups showed statistically significant differences compared to the control group (all *p* < 0.05).

## 4. Discussion

This study revealed that asthma is associated with periodontitis and that participants with well-controlled asthma have the highest ORs (2.15) for periodontitis in the Korean adult population. The results can be explained by delineating the reciprocal relationship between asthma and periodontitis.

A positive association between asthma and periodontitis was observed in the present study. Aligning with these findings, several studies point to a link between asthma and periodontal disease [16,17,18,23], although some studies have reported opposite results [14,15,24]. A case–control study in Brazil noted an association between periodontitis and severe asthma [16], and a population-based study in Taiwan showed an elevated risk of developing periodontal disease in patients with asthma [17]. In a Korean population-based study, patients with a current asthma condition were approximately five times more likely to be associated with periodontitis [18]. Moreover, a recent meta-analysis of six studies, capable of quantitative synthesis, highlighted that patients with asthma present more periodontal disease compared to healthy individuals [23]. The meta-analysis results of the calculus index (mean difference = 0.57, 95% CI = 0.44–0.70), papillary bleeding index (mean difference = 0.72, 95% CI = 0.52–0.93), and clinical attachment loss (mean difference = 1.13, 95% CI = 0.09–2.18) demonstrated higher means for the asthmatic group. Another systematic review and meta-analysis also indicated statistically significant differences for the parameters of gingival bleeding (mean difference = 0.70, 95% CI = 0.64–0.75), plaque index (mean difference = 0.43, 95% CI = 0.32–0.53), and gingival index (mean difference = 0.51, 95% CI = 0.22–0.80) in participants with asthma [25]. Conversely, a cross-sectional study in the United States reported a significant inverse association between self-reported asthma and periodontal disease [14], and a prior study in Austria showed that patients with periodontitis had a significantly lower prevalence of asthma than the general population [15]. In a recent population-based observational study in the United States, current-asthmatic adults were less likely to have severe periodontitis compared with never-asthmatic adults (adjusted OR = 0.51, 95% CI = 0.30–0.87 as classified using Centers for Disease Control and American Academy of Periodontology (CDC/AAP) case definition and adjusted OR = 0.58, 95% CI = 0.35–0.97 as classified using the Periodontal Profile Class System, respectively) [24]. As conflicting results are being reported, a population-based study using a large sample size, such as the present study, can be considered important.

Several factors related to asthma are associated with the development of periodontitis. Firstly, salivary flow, crucial for oral health, may be compromised in patients with asthma due to the relevant medications, diet, or lifestyle [26]. Long-acting β2-agonists, inhaled corticosteroids, and antihistamines are known to reduce salivary secretion and alter salivary composition [27]. Reduced salivary flow creates a conducive environment for the growth and proliferation of microorganisms such as *Streptococcus mutans* and *Lactobacillus* spp., which can cause dental caries [28]. Secondly, patients with asthma have an increased tendency to switch to mouth breathing [29]. Mouth breathing can lead to dehydration of the alveolar mucosa and increased gingival inflammation, owing to reduced epithelial resistance to bacterial plaque [30,31]. Lastly, a recent study reported that patients with asthma show a more pronounced microbial shift than healthy controls, suggesting an association between asthma and oral bacteria [32].

Pathogens linked to periodontitis may play a role in asthma development. Increasing evidence has shown that oral microecosystems, comprising the oral microbiome, saliva, and interactions between the oral microbiota and host, are linked to respiratory diseases, including asthma [33]. Various respiratory pathogens have been detected in dental plaque, periodontal pockets, and saliva, indicating that the oral microecosystem may serve as a potential source of respiratory diseases [34]. There have been a few reports of the oral microbiota being associated with the development of allergies and asthma [35,36]. Recent studies have reported that periodontal disease is a potential risk factor for systemic inflammatory diseases, including respiratory disease [37,38,39]. This correlation may be attributed to inflammatory and immunological responses common to both periodontal disease and systemic diseases, leading to an increase in circulating inflammatory cytokines, such as C-reactive protein (CRP), interleukin (IL)-6, and tumor necrosis factor (TNF)-α [40].

In the present study, data were collected only from participants aged >40 years. Previous studies have shown that aging is a significant risk factor for chronic diseases, including autoimmune, infectious, and inflammatory diseases such as periodontitis. Aging alone results in the physiological loss of periodontal attachment and alveolar bone. To evaluate the association between asthma and periodontitis regardless of the effect of aging, we excluded the young adult group. Furthermore, in the subgroup analysis, age groups were divided into <53 years old and ≥53 years old, but the relationship between asthma and periodontitis was consistent.

Our findings indicate that patients with well-controlled asthma have the highest ORs for periodontitis. In addition, the ‘being treated’ asthma subgroup did not show statistical significance compared to the control in all the men, and in the <53-year-old women. This finding aligns with the results of a cross-sectional study using the 2014 Korea National Health and Nutrition Examination Survey data, which demonstrated that patients taking scheduled anti-asthmatic medications were less likely to be diagnosed with periodontitis [18]. This could be because anti-asthmatic medications, such as inhaled corticosteroids, leukotriene modifiers, and long-acting β2-agonists, may inhibit or modify the proinflammatory response. However, contrary to our results, a Taiwanese population study showed that patients receiving inhaled corticosteroid therapy have greater hazard ratios for periodontal diseases than non-users. They explained that inhaled corticosteroids may reduce bone mineral density and lead to systemic bone loss associated with periodontal diseases [17]. Future studies will be required to elaborate in detail the exact mechanisms underlying the differences between anti-asthmatic medications in the association of asthma with periodontitis. In addition, the ‘being treated’ patients with asthma may have received more attention for dental care and resolved their dental problems earlier. This could be because they receive regular dental care while visiting the clinic for anti-asthmatic medications.

The present study has several limitations. Firstly, the history of periodontitis and asthma relied on self-reported questionnaires, potentially introducing surveillance or recall bias. Disease diagnosis methods using questionnaires are widely accepted in large population-based studies. In addition, dental examinations are usually performed in most Koreans through regular visits guaranteed by the National Health Insurance, owing to the advantages of cost-effectiveness, widespread coverage, and easy access to medical institutions in Korea. Secondly, it was not possible to assess which drugs the patients were using, such as oral or inhaled corticosteroids. However, the individuals with asthma were categorized into the ‘well-controlled’, ‘being treated’, and ‘not being treated’ groups. Our results suggest that patients with untreated asthma face a heightened risk of periodontitis compared to those taking medications. Finally, due to the unavailability of information on dental examinations and lung function tests, the severity of both periodontitis and asthma remained unknown. Considering the constraints of this study, further research, including pulmonary function test results for the diagnosis of asthma, dental examination results for the severity of periodontitis, and more detailed information on the medical treatment method, are warranted to further validate the current results. However, in the present study, the utilization of a large Korean population augmented the statistical robustness, enabling the generalization of results to the overall population. Additionally, we adjusted for various covariates, encompassing nutritional intake and income, which influence both asthma and periodontitis. 

## 5. Conclusions

The current study unveiled an association between asthma and periodontitis in Korean adults. Patients with well-controlled asthma showed the highest ORs for periodontitis. Clinicians should be aware of the risk of periodontitis in patients with asthma, even those with well-controlled asthma.

## Figures and Tables

**Table 1:** General characteristics of participants.

Characteristics		Total Participants		
		Asthma	Control	*p*-Value
Age (mean, SD, y)		55.6 (8.6)	53.0 (8.3)	<0.001 *
Sex, *n* (%)				<0.001 *
	Male	747 (29.6)	46,521 (35.0)	
	Female	1774 (70.4)	86,285 (65.0)	
BMI (mean, SD, kg/m^2^)		24.4 (3.3)	23.9 (2.9)	<0.001 *
Income (*n*, %)				<0.001 *
	Missing, no response	248 (9.8)	11,377 (8.6)	
	Lowest	922 (36.6)	38,112 (28.7)	
	Middle	842 (33.4)	52,267 (39.4)	
	Highest	509 (20.2)	31,050 (23.4)	
Smoking status (*n*, %)				<0.001 *
	Non-smoker	1862 (73.9)	95,972 (72.3)	
	Former smoker	406 (16.1)	19,986 (15.0)	
	Current smoker	253 (10.0)	16,848 (12.7)	
Alcohol consumption (*n*, %)				<0.001 *
	Non-drinker	1444 (57.3)	67,397 (50.7)	
	Former drinker	129 (5.1)	4887 (3.7)	
	Current drinker	948 (37.6)	60,522 (45.6)	
Nutritional intake				
	Total calories (kcal/d)	1728.9 (574.5)	1750.6 (570.2)	0.058
	Protein (g/d)	58.4 (25.8)	59.7 (26.5)	0.002 *
	Fat (g/d)	27.0 (17.5)	28.3 (18.3)	<0.001 *
	Carbohydrate (g/d)	309.2 (95.8)	310.2 (92.9)	0.591
Periodontitis (*n*, %)		330 (13.1)	9655 (7.3)	<0.001 *

SD = standard deviation; BMI = body mass index. * Independent t-test or Chi-square test. Significance at *p* < 0.05.

**Table 2:** Crude and adjusted odds ratios (95% confidence interval) for periodontitis in asthma and control groups.

Characteristics		Odds Ratios for Periodontitis			
		Crude	*p*-Value	Adjusted ^†^	*p*-Value
Total participants (*n* = 135,327)					
	Asthma	1.92 (1.71–2.16)	<0.001 *	1.79 (1.59–2.02)	<0.001 *
	Control	1.00		1.00	
Age < 53 years old, men (*n* = 21,514)					
	Asthma	2.18 (1.49–3.19)	<0.001 *	2.17 (1.48–3.20)	<0.001 *
	Control	1.00		1.00	
Age < 53 years old, women (*n* = 45,465)					
	Asthma	1.73 (1.34–2.23)	<0.001 *	1.64 (1.27–2.12)	<0.001 *
	Control	1.00		1.00	
Age ≥ 53 years old, men (*n* = 25,754)					
	Asthma	1.83 (1.44–2.33)	<0.001 *	1.87 (1.47–2.38)	<0.001 *
	Control	1.00		1.00	
Age ≥ 53 years old, women (*n* = 42,594)					
	Asthma	1.87 (1.57–2.24)	<0.001 *	1.81 (1.51–2.16)	<0.001 *
	Control	1.00		1.00	

* Logistic regression model, significance at *p* < 0.05. ^†^ Models adjusted for age, sex, income group, body mass index, smoking, alcohol consumption, and nutritional intake (total calories, protein, fat, and carbohydrate intake).

**Table 3:** Crude and adjusted odds ratios (95% confidence interval) for periodontitis according to the current status of asthma management.

Characteristics		Odds Ratios for Periodontitis			
		Crude	*p*-Value	Adjusted ^†^	*p*-Value
Total participants (*n* = 128,255)					
	Asthma well-controlled	2.16 (1.69–2.78)	<0.001 *	2.15 (1.68–2.76)	<0.001 *
	Asthma being treated	1.62 (1.31–2.01)	<0.001 *	1.44 (1.16–1.78)	<0.001 *
	Asthma not being treated	1.94 (1.62–2.32)	<0.001 *	1.86 (1.55–2.22)	<0.001 *
	Control	1.00		1.00	
Age < 53 years old, men (*n* = 21,514)					
	Asthma well-controlled	2.33 (0.98–5.54)	0.056	2.54 (1.06–6.10)	0.037 *
	Asthma being treated	1.84 (0.88–3.86)	0.106	1.73 (0.82–3.64)	0.150
	Asthma not being treated	2.29 (1.34–3.89)	0.002 *	2.31 (1.35–3.95)	0.002 *
	Control	1.00		1.00	
Age < 53 years old, women (*n* = 43,219)					
	Asthma well-controlled	2.07 (1.31–3.27)	0.002 *	2.01 (1.27–3.17)	0.003 *
	Asthma being treated	1.43 (0.83–2.48)	0.199	1.32 (0.76–2.29)	0.321
	Asthma not being treated	1.54 (1.05–2.26)	0.027 *	1.49 (1.05–2.19)	0.041 *
	Control	1.00		1.00	
Age ≥ 53 years old, men (*n* = 24,519)					
	Asthma well-controlled	1.69 (0.91–3.12)	0.096	1.80 (0.97–3.34)	0.062
	Asthma being treated	1.29 (0.86–1.95)	0.221	1.32 (0.87–1.99)	0.192
	Asthma not being treated	2.75 (1.93–3.92)	<0.001 *	2.75 (1.93–3.92)	<0.001 *
	Control	1.00		1.00	
Age ≥ 53 years old, women (*n* = 40,317)					
	Asthma well-controlled	2.48 (1.72–3.59)	<0.001 *	2.41 (1.67–3.49)	<0.001 *
	Asthma being treated	1.62 (1.20–2.22)	0.002	1.55 (1.14–2.11)	0.005 *
	Asthma not being treated	1.71 (1.29–2.26)	<0.001 *	1.66 (1.25–2.20)	<0.001 *
	Control	1.00		1.00	

* Logistic regression model, significance at *p* < 0.05. ^†^ Models adjusted for age, sex, income group, body mass index, smoking, alcohol consumption, and nutritional intake (total calories, protein, fat, and carbohydrate intake).

## Data Availability

The data in this study were from the Korean National Health Insurance Service-Health Screening Cohort. Releasing of the data by the researcher is not allowed legally. All of the data are available from the database of the National Health Insurance Sharing Service (NHISS) (https://nhiss.nhis.or.kr/) (accessed on 12 July 2021). NHISS allows all data at a cost for any researcher who promises to follow the research ethics. Anyone wanting to access the data for this article can download it from the website after promising to follow the research ethics.
